# Antibiotic-Prescribing Habits in Dentistry: A Questionnaire-Based Study

**DOI:** 10.3390/antibiotics13020189

**Published:** 2024-02-16

**Authors:** Luca Sbricoli, Giulio Grisolia, Edoardo Stellini, Christian Bacci, Marco Annunziata, Eriberto Bressan

**Affiliations:** 1 Department of Neurosciences, Unit of Dentistry, University of Padova, 35122 Padova, Italy; luca.sbricoli@unipd.it (L.S.); giulio.grisolia@studenti.unipd.it (G.G.); edoardo.stellini@unipd.it (E.S.); christian.bacci@unipd.it (C.B.); eriberto.bressan@unipd.it (E.B.); 2 Multidisciplinary Department of Medical-Surgical and Dental Specialties, University of Campania Luigi Vanvitelli, 80138 Naples, Italy

**Keywords:** antibiotics, prophylaxis, implant dentistry

## Abstract

The problem of antibiotic resistance is becoming increasingly serious worldwide due to uncontrolled prescription. Dentists are among the groups that prescribe the most antibiotics, often to delay urgent treatment. The purpose of the present study is to investigate the prescribing protocols adopted by dentists for prophylaxis and antibiotic therapy in major clinical surgical indications. Methods: A ten-question survey was administered to a group of Italian dentists. The participants were asked about their preferences for antibiotic administration for the prevention of infective endocarditis, the administration of antibiotics to patients allergic to penicillin, the insertion of implants, and the extraction of third molars. The retrieved data were screened and analyzed. Results: A total of 298 surveys were filled out. The most-prescribed antibiotic was amoxicillin or amoxicillin with clavulanic acid or macrolides for allergic patients. The administration of two grams of amoxicillin one hour before surgery was the most widely used prescriptive protocol for prophylaxis. International guidelines on antibiotic prophylaxis for infective endocarditis were only partially followed. The most heterogeneous results emerged for prophylaxis associated with dental implants or provided prior to surgical third-molar extraction. Conclusions: The present study shows widespread antibiotic prescriptive heterogeneity among the sample of dentists analyzed, especially in conditions where international guidelines are lacking. An evidence-based consensus on prescriptive modalities in dentistry would be desirable in the near future.

## 1. Introduction

One of the major risks resulting from the overuse of antibiotics is the development of antibiotic resistance (AR), i.e., the ability of microorganisms to withstand antimicrobial treatment thanks to the development of specific defense mechanisms [1]. In the last decade, there has been an increase in AR, which currently poses a significant and growing public health concern worldwide. Recent data have shown that 4.95 million deaths were associated with AR and 1.27 million deaths were caused by AR in 2019 [2]. The evolution of AR reduces available therapies and has a heavy clinical and economic impact. In addition, the continued inappropriate use of antibiotics promotes the spread of this phenomenon and resistant pathogens. For these reasons, any antibiotic prescription should be given according to well-established protocols, clinical recommendations, and guidelines either for therapy or prophylaxis.

Antibiotic therapy (AT) refers to the administration of antibiotics for the purpose of pharmacologically counteracting an ongoing infection. It should be prescribed according to the site and type of an infection, aided by bacteriological tests and related antibiograms when possible, and continued at appropriate doses and until the infection is completely cured [3].

Antibiotic prophylaxis (AP) refers to the administration of antibiotics in the absence of an infection, with the purpose of preventing its onset and spread. Therefore, AP is not prescribed for therapeutic purposes, and it should be even more conscientiously and carefully administered.

Dentists are responsible for about 10% of antibiotic prescriptions for humans worldwide, either as prophylaxis or therapy [4], but up to 80% of antibiotics prescribed before dental procedures in order to prevent infection are considered unnecessary [5,6].

In dentistry, the most commonly prescribed antibiotic is penicillin, especially amoxicillin, which is often combined with clavulanic acid [7,8]. This is due to its broad spectrum, wide availability, and reduced interactions with other drugs. For patients allergic to the above molecules, macrolides are predominantly used.

Several systematic reviews recommend the oral administration of 2 g of an antibiotic, in a single dose, about one hour before extractive and/or dental implant surgery and report no significant benefits regarding the continuation of antibiotic administration in the following days [8]. Currently, however, there are no guidelines on antibiotic prescription before dental implant and extractive surgery.

The only guidelines in the literature regarding AP were developed by the American and European cardiologists’ associations and concern the prevention of infective endocarditis (IE) [9,10].

The purpose of the present questionnaire-based study is to gain an insight into the antibiotic-prescribing habits of Italian dentists either for AP or full AT. In other words, do this area’s dentists know the guidelines and follow them when prescribing antibiotics in the most common clinical situations?

## 2. Results

A sample of 298 Italian dentists answered the survey (response rate: 43.8%). The demographic characteristics of the sample are summarized in Table 1. Briefly, most of the respondents were men (*n* = 175; 58.7%), graduated in Italy (*n* = 290; 96.6%), and worked in a private practice (*n* = 291; 97.7%), and almost half of the sample consisted of individuals ≤40 years old (*n* = 158; 53%). The most prescribed antibiotic was Amoxicillin + clavulanic acid (*n* = 188; 63.1%), followed by Amoxicillin (*n* = 106; 35.6%) (Table 2). The majority of dentists younger than 50 years preferred the combination of amoxicillin + clavulanic acid to amoxicillin alone as the first-choice antibiotic (132 (64%) and 73 (36%) dentists, respectively). Similar results emerged for older dentists: 56 (63%) preferred the combination of amoxicillin + clavulanic acid, while 33 (37%) preferred amoxicillin alone. More than three-quarters of the sample (*n* = 242; 81.2%) declared that the administration of 2 g of Amoxicillin 1 h before surgery was their preferred protocol for AP in nearly half of the cases associated with clavulanic acid. Another suggested protocol for AP consisted of the administration of 1 g of Amoxicillin or Amoxicillin + clavulanic acid 1 h before (10%) or starting from 1 day before (1.3%) treatment, whereas 7% prolonged AP for up to six days after treatment (7%) (Table 3). When dealing with patients allergic to penicillin, 82% of the dentists declared they prescribed a macrolide, usually Clarithromycin (*n* = 123; 41.2%) (Table 4).

A wide variety of responses emerged regarding the indications for IE prophylaxis (Table 5). The vast majority of the dentists prescribed prophylaxis for patients with a history of IE (*n* = 237; 80%), with valve prostheses/repairs (*n* = 186; 62%), and with cyanotic heart disease (*n* = 147; 49%). A considerable number of dentists considered prophylaxis in cases of a cardiac transplant (*n* = 190; 64%), a valve pathology (*n* = 134; 45%), an immunocompromised patient (*n* = 94; 32%), and a patient with a pacemaker or defibrillator (*n* = 40; 13%). Only a small percentage of dentists never prescribe AP (*n* = 13; 4%) or, in contrast, always prescribe antibiotics for any type of prior or current cardiac disease (*n* = 23; 8%).

Almost half of the participants who prescribe antibiotics in the case of implant insertion in ASA 1 patients (Table 6) advise their patients to undergo the full course of therapy (*n* = 152; 51%). Ninety-three dentists (31%) prescribe only AP, and fifty-three dentists (18%) never prescribe antibiotics. Slight differences emerged between age groups: younger dentists reported prescribing the full course of antibiotics in 45.9% of cases, which can be compared with 62.9% of older dentists. Similar percentages of dentists reported prescribing prophylaxis alone (32.5% of younger dentists versus 28% of older dentists). In total, 21.5% of younger dentists reported choosing not to prescribe antibiotics, while 9% of older dentists reported engaging in this behavior.

The same percentage of colleagues not prescribing antibiotics in the case of implant insertion (18%) did not prescribe antibiotics in the case of mandibular third-molar extraction either (Table 7). The remaining participants always prescribe antibiotics, but with some differences: thirty-seven dentists (12%) prescribe only prophylaxis, one hundred and fifteen (39%) prescribe the full therapy, and ninety-two (31%) regularly prescribe prophylaxis but suggest the full therapy in case of ostectomy. Younger dentists reported prescribing prophylaxis alone in 13.9% of cases, prescribing the full course of antibiotics in 39% of cases, and not prescribing antibiotics in 20.6% of cases. Similar percentages emerged from the answers given by older dentists: 12.4% of dentists declared they do not prescribe antibiotics, 9% prescribe only prophylaxis, and 48.3% prescribe the entire course of antibiotic therapy.

## 3. Discussion

The present study investigated antibiotic-prescribing habits in common dental situations among a sample of Italian dentists. The data from the analysis showed wide prescriptive heterogeneity and a non-compliant adherence to international antibiotic-prescribing guidelines.

One of the most controversial aspects concerns prophylaxis for IE. The available international guidelines, American [10] and European [9], agree that there is a modest degree of evidence with which to recommend the administration of AP in cases of the presence of any heart valve prosthesis or repairs, in patients who have experienced previous episodes of IE, and in cases of patients with cyanotic heart defects. In the present study, it was found that only a portion of the dentists adhere to these guidelines, while a considerable percentage of colleagues improperly prescribe antibiotics, both in terms of active principle and indications. Amoxicillin + clavulanic acid is prescribed almost twice as often as Amoxicillin. It is difficult to identify a clinical rationale behind this preference towards the association between amoxicillin and clavulanic acid, in which 1750 milligrams of amoxicillin is administered to a patient instead of 2000 milligrams. A possible explanation lies in a clinical practice based on personal experience provided through advice given by older and more experienced colleagues rather than clinical use based on the study of guidelines. Furthermore, AP for the prevention of IE is frequently prescribed for patients with a pacemaker or defibrillator, immunocompromised patients, cardiac transplant patients, and patients with valve pathologies, who may be widely diffused in the population. A mitral valve prolapse, for example, represents the most common cardiac malformation in developed countries, with a prevalence between 1 and 2.5 percent [11]. In most cases, it is asymptomatic and diagnosed during sports medicine fitness examinations. Surprisingly, the same patients who present with this abnormality are accustomed to taking prophylactic antibiotics before dental sessions on recommendation of cardiologists or general practitioners. This habit has led, over the years, to the administration of AP being considered routine even for dentists, when the opposite would, in fact, be desirable in order to reduce the risk of AR. Nowadays, the range of indications for antibiotic prophylaxis in the case of infective endocarditis is increasingly narrow [9,10]. If the trend towards an excessively generous prescribing habit does not reverse, there is a real risk that antibiotics will become less and less effective against bacteria, putting more and more patients at risk of infective endocarditis.

Another interesting aspect that emerged from the present work is the behavior in the case of dental implant placement in ASA 1 patients. The questionnaire revealed that more than half of the sample prescribes the entire course of AT. It must be noted, however, that the survey questions do not distinguish between single and multiple implants. A significant percentage of participants could prescribe the entire AT course only in the case of multiple implants but not for a single implant. Therefore, there may have been an overestimation of the final results. It must be underlined that several systematic reviews of the literature do not support this type of administration (entire course of AT) in case of implant placement. Specifically, Esposito et al. [12] showed a positive effect of AP alone on implant survival. Similar results were found by Lund et al. [13] and Romandini et al. [14], suggesting a reduction in early implant failure with the administration of AP alone. Finally, a recent systematic review further confirmed the effectiveness of antibiotic administration in preventing early implant failure, declaring that AP is most likely the best protocol [15]. Thus, increasing evidence supports AP instead of a complete lack of antibiotic administration or prescribing the full therapeutic dose, and it would be desirable for the dentists to have clinical guidelines concerning this indication for their daily practice. 

A similar argument can be made for antibiotic prescription in cases of mandibular third-molar extraction. In fact, the vast majority of respondents reported administering antibiotics, with a clear prevalence for full therapy over prophylaxis, especially in cases requiring ostectomy. A 2016 systematic review with a meta-analysis concluded that AP alone reduces the risk of alveolitis and infection in 70% of cases of surgical third-molar extractions [16], while a more recent Cochrane systematic review stated that simple antibiotic administration versus a placebo was able to reduce infection and alveolitis in 66% and 34% of cases, respectively [17]. The latter review, however, did not reach a definitive conclusion on the best timing of administration. A very recent network meta-analysis [18], exclusively focusing on lower-third-molar extraction and including sixteen RCTs conducted on 2158 patients and 2428 extractions, found that the use of antibiotic prophylaxis significantly reduced the number of dry-socket and surgical site infection events (OR 0.54 and 0.36, respectively), but with a high number of patients requiring treatment, namely, 25 and 18, respectively, thus recommending caution in the prescribing habit with this indication.

The stratification of the data by age revealed an interesting and promising finding: younger dentists (less than 50 years old) tend to prescribe fewer antibiotics than older dentists. In fact, in the case of implant placement in ASA 1 patients, 45.9 percent of younger dentists prescribe the full course of antibiotic therapy, which can be compared to 62.9 percent of older dentists, a gap close to 20 percent. As a result, more than 50% of younger dentists tend not to prescribe antibiotics or prescribe only prophylaxis, showing a positive trend toward greater caution in antibiotic prescribing.

The same trend was found, although to a lesser extent, in prescribing habits in cases of mandibular third-molar extraction. The rates of a complete lack of antibiotic prescription or prophylaxis-only administration were between 20 and 30 percent among the dentists. The interesting aspect of these data is that dentists seem to be much more concerned about infections and complications following mandibular third-molar extraction than implant placement, with a substantial difference in the antibiotic prescription regimens.

On the other hand, regarding the type of antibiotic used for AP, stratification by age did not show significantly different trends between young and adult dentists. The prescribing rates were almost overlapping, with a clear preference toward the combination of amoxicillin + clavulanic acid (corresponding to just over 60 percent of the respondents). This finding contrasts with antibiotic-prescribing guidelines, as available indications recommend the use of amoxicillin alone, not combined with clavulanic acid, which results in the administration of 1750 milligrams of amoxicillin, while the recommended does is 2000 milligrams.

Finally, an interesting aspect that emerged from the analysis of the responses concerns clindamycin. This type of antibiotic, belonging to the lincosamide class, was described by 24 dentists (8%) as an alternative for patients allergic to amoxicillin. Only the most recent versions of the AP guidelines have specified that clindamycin is no longer recommended for AP in dental procedures. This might suggest that dentists are unaware of the most up-to-date versions of the guidelines, and the present paper is intended to provide a useful cue for professional development toward more informed antibiotic administration.

Regarding the generalizability of the present study, which is probably the greatest limitation of the present paper, it can be said that the sample, although limited to a specific Italian region, is wider than that of any previously published paper on the same topic [19,20] and is conditioned by the response rate for the survey. Although a response rate of 43.8% is an excellent figure for a survey according to recent studies [21], such a rate can undoubtably influence the generalizability of a study. In this sense, an electronic survey was preferred in order to allow simple and immediate answers to the survey, exploiting its user-friendly interface. Furthermore, it facilitates data collection and analysis, avoiding errors in reporting data from a paper questionnaire on an electronic database.

Data from previously published papers on the same topic [19,20] are partially in agreement with the present study, but an promising trend can be derived: the tendency to not prescribe antibiotics, both in the case of implant surgery and in the case of wisdom tooth extraction, is increasing (18% vs. 14.8% and 1% for implant surgery in Lollobrigida et al.’s and Rodriguez-Sanchez et al.’s studies and 18% vs. 7.7% and 14.8% for wisdom tooth extraction). The tendency to prescribe the entire AT compared to AP alone or non-prescription is also maintained in studies on populations of other countries, with percentages between 2.1% and 10% for the non-prescribing group [7,22]. Nevertheless, it should be noted that the present study describes the prescribing habits of dentists from private practice since the Italian dentistry activity is mostly private.

The findings of the present study increase the consistency of data on dentists’ prescribing habits. In clinical situations that require an antibiotic prescription, clinicians should evaluate the existence of dedicated guidelines and prescribe antibiotics based on the indications. This behavior will certainly have general repercussions on the population and on the fight against antibiotic resistance—an increasingly relevant problem.

## 4. Materials and Methods

### 4.1. Study Design

This is a self-reported questionnaire-based research work. The document consisted of ten questions, seven of which were single choice with only one possible answer and three open-ended questions. The questionnaire (Supplementary File) was administered via an electronic form (https://docs.google.com/forms, accessed on 1 October 2021) to a mailing list of dentists in the province of Padua (Italy) (*n* = 680). The list was obtained from an electronic database of dentists who graduated from the University of Padua. The survey was completely anonymous.

### 4.2. Questionnaire

The first four questions addressed demographic information such as gender, age, place of graduation, and employment status. In the fifth question, the participants were asked to select the most-prescribed antibiotic. Question number six asked the participants to specify the AP protocol adopted for non-penicillin-allergic patients. Question number seven asked what kind of antibiotic should be prescribed to penicillin-allergic patients. Question number eight asked participants which patients should be prescribed as AP before a professional dental hygiene procedure. In question number 9, the candidates were asked whether he/she prescribes AP and/or AT in case of conventional dental implant surgery for a healthy patient, in which one or more implants are placed, without hard or soft tissue regeneration. In question number 10, the candidates were asked whether he/she prescribes AP and/or AT in the case of the surgical extraction of a mandibular included wisdom tooth in a healthy patient, in which a flap, possible ostectomy, and/or odontotomy is performed. The online survey did not allow for blank answers.

### 4.3. Data Analysis

The data obtained from the online questionnaire were transferred to a spreadsheet for evaluation. In the case of open-ended questions, answers were aggregated into common categories for analysis. Some of the answers were also categorized by age. The cut-off was set at 50 years old. Data were then analyzed using descriptive statistics. The software used was Microsoft Excel version 16.78 (Microsoft Corporation, Redmond, WA, USA).

## 5. Conclusions

The present study shows widespread antibiotic prescriptive heterogeneity among the sample of dentists analyzed, especially in conditions where international guidelines are lacking. An evidence-based consensus on prescriptive modalities in dentistry would be desirable in the near future. Pending further research, clinicians are strongly encouraged to follow the available clinical practice guidelines and base their clinical decisions about antibiotic prescription on the scientific evidence and common sense in order to preserve patients’ health, limiting, on the one hand, the risks of post-operative infections and, on the other hand, the ever-increasing risk of AR. This study adds consistent numerical data on dentists’ prescribing habits to help raise awareness among colleagues and thus promote more-informed prescription of antibiotics.

Based on the published evidence, the following clinical guidelines and recommendations can be provided:

- Clinical guidelines are available {Citation} [9] indicating that AP for the prevention of IE is recommended only in cases where there is “manipulation of gingival tissue, manipulation of the periapical region of teeth, or perforation of the oral mucosa” in patients with previous IE; patients with a surgically implanted valve, transcatheter valve, or prior valve repair; patients with uncorrected cyanotic congenital heart disease (CHD) or those with CHD and prior repair involving prosthetic material; and patients with a ventricular assistance device. The recommended antibiotics for oral consumption 30–60 min before procedure are amoxicillin (2 g for adults, 50 mg/kg for children) for non-allergic patients and Cephalexina (2 g for adults 50 mg/kg orally for children); Azithromycin or clarithromycin (500 mg for adults, 15 mg/kg for children); or Doxycycline (100 mg for adults and >45 kg children, 2.2 mg/kg for <45 kg children).
- No clinical guidelines are available, but there is increasing evidence showing that the administration of antibiotics can reduce the risk of alveolitis and post-operative infections in surgical extractions of impacted third molars.
- No clinical guidelines are available, but there is increasing evidence that the administration of antibiotics may prevent early implant failure due to operative site infection.
- In the absence of clinical guidelines, clinicians should evaluate the need to prescribe antibiotics for each individual patient, taking into consideration the presence of systemic conditions and the case-specific risk of developing a postoperative infection.

## Figures and Tables

**Table 1:** Demographic characteristics of the sample.

Personal Characteristics	Number	Percentage
SEX		
Female	123	41.3%
Male	175	58.7%
AGE (YEARS)		
21–30	82	27.5%
31–40	76	25.5%
41–50	58	19.5%
51–60	40	13.4%
61–70	42	14.1%
PLACE OF GRADUATION		
Italy	290	97.3%
Spain	6	2%
Portugal	1	<1%
Romania	1	<1%
EMPLOYMENT STATUS		
Private practice	291	97.7%
Public health system	7	2.3%

**Table 2:** Most-prescribed antibiotics among participants (either AT or AP).

Active Principle	Total	≤50 Years	>50 Years
Amoxicillin	106 (35.6%)	73 (36%)	33 (37%)
Amoxicillin + clavulanic acid	188 (63.1%)	132 (64%)	56 (63%)
Bacampicillin	1 (<1%)	<1%	-
Claritromicin	2 (<1%)	<1%	-
Doxycycline	1 (<1%)	<1%	-

**Table 3:** Antibiotic schemes intended to serve as prophylaxis reported by the respondents. Amox = amoxicillin; h = hour; g = gram.

Antibiotic Scheme	Number	Percentage
1 g Amox 1 h before	13	4.3%
1 g Amox + clavulanic acid 1 h before	17	5.7%
2 g Amox 1 h before	137	46%
2 g Amox + clavulanic acid 1 h before	105	35.2%
1 g Amox the day before + 1 g Amox 1 h before	4	1.3%
1 g Amox 1 h before + 1 g every 12 h for six days	8	2.6%
1 g Amox + clavulanic acid 1 h before + 1 g every 12 h for six days	13	4.3%
Other	1	<1%

**Table 4:** Alternative antibiotics for penicillin-allergic patients.

Drug Class (Active Principle)	Total
Macrolides (Azithromycin, Clarithromycin, Erythromycin)	243 (81.6%)
Cephalosporins (Ceftriaxone)	10 (3.4%)
Fluoroquinolones (Ciprofloxacin)	3 (1%)
Lincosamides (Clindamicin)	24 (8%)
Others	6 (2%)
Respondent did not know	12 (4%)

**Table 5:** Indications for administering AP to prevent IE before professional dental hygiene procedure.

Indication for AP to Prevent IE	Number	Percentage
Always	23	8%
Never	13	4%
History of endocarditis	237	80%
Heart transplant	190	64%
Valve prostheses or valve repair	186	62%
Cyanotic heart disease	147	49%
Valve pathology	134	45%
Immunocompromised patients	94	32%
Patients with pacemaker or defibrillator	40	13%
Others	3	1%

**Table 6:** Antibiotic prescription in the case of dental implant placement in ASA 1 patient.

Antibiotic Prescription in Case of Dental Implant Placement	Total	≤50 Years	>50 Years
No antibiotic	53 (18%)	45 (21.5%)	8 (9%)
Only prophylaxis	93 (31%)	68 (32.5%)	25 (28%)
Full therapy	152 (51%)	96 (45,9%)	56 (62.9%)

**Table 7:** Antibiotic prescription in case of mandibular third-molar extraction.

Antibiotic Prescription in Case of Mandibular Third-Molar Extraction	Total	≤50 Years	>50 Years
No antibiotics	54 (18%)	43 (20.6%)	11 (12.4%)
Only prophylaxis	37 (12%)	29 (13.9%)	8 (9%)
Full therapy	115 (39%)	72 (34.4%)	43 (48.3%)
Prophylaxis + full therapy only in case of ostectomy	92 (31%)	65 (31.1%)	27 (30.3%)

## Data Availability

Data are available upon request made to the corresponding authors.

## Supplementary Materials

The following supporting information can be downloaded at: https://www.mdpi.com/article/10.3390/antibiotics13020189/s1, Supplementary File: Questionnaire for Antibiotic Prophylaxis Protocol.
