# Supplementary material for: Antibiotic-Prescribing Habits in Dentistry: A Questionnaire-Based Study
Source: Antibiotics (Basel). 2024 Feb 16;13(2):189. doi: 10.3390/antibiotics13020189 (PMC10886335; doi:10.3390/antibiotics13020189)
Supplement: Supplementary file 1 [file antibiotics-13-00189-s001.zip › antibiotics-2847424-supplementary.pdf]

QUESTIONNAIRE FOR ANTIBIOTIC PROPHYLAXIS PROTOCOL

Dear colleague,

We kindly ask you to fill the present survey about antibiotic prescription habits in dentistry. The questionnaire is completely anonymous, so please answer honestly, according to your daily clinical practice.

1. Gender:

- o M
- o F

2.Age:

- o 21-30
- o 31-40
- o 41-50
- o 51-60
- o 61-70
- o +70

3.Graduation country:

- o Italy
- o Spain
- o Portugal
- o Other ________________

4.Employment status:

- o Private practice
- o National Health System (NHS)

5. What is your favorite antibiotic (what you prescribe/have prescribed more)?

____

6. What do you mean by antibiotic **<u>PROPHYLAXIS</u>** in a penicillin non-allergic patient?

- o 1 g of Amoxicillin 1 hour before
- o 1 g of Amoxicillin + Clavulanic Acid 1 hour before
- o 2 g of Amoxicillin 1 hour before
- o 2 g of Amoxicillin + Clavulanic Acid 1 hour before
- o 3 g of Amoxicillin 1 hour before
- o 3 g of Amoxicillin + Clavulanic Acid 1 hour before
- o 1 g of Amoxicillin starting the day before + 1 g the same morning
- o 1 g of Amoxicillin + Clavulanic Acid starting the day before + 1 g the same morning
- o 1 g of Amoxicillin 1 hour before and then 1 g every 12 hours for 6 days
- o 1 g of Amoxicillin + Clavulanic Acid 1 hour before and then 1 g every 12 hours for 6 days
- o Other ____

7. Which antibiotic do you prescribe in case of penicillin allergic patients?

____

8. In case of professional dental hygiene, in which cases do you think antibiotic prophylaxis is useful for bacterial endocarditis? (More answers possible)

- Always (for any current or prior cardiac disease)
- Never
- Patients with pacemaker or defibrillator
- Immunosuppressed patients
- Patients with valve pathology
- Patients with valve prosthesis or repair
- Patients with a history of endocarditis
- Patients with cyanotic heart disease
- Heart transplant patients
- Other ______________________________

9. In case of 1 or more implants placement **in a healthy patient,** without hard and soft tissue regeneration (conventional implant surgery):

- You don't prescribe antibiotics
- You prescribe only antibiotic prophylaxis, as indicated in question number 6
- You prescribe full antibiotic therapy

10. In case of surgical extraction of impacted mandibular wisdom tooth **in healthy patient** (flap raising, optional ostectomy and odontotomy):

- You don't prescribe antibiotics
- You prescribe only antibiotic prophylaxis
- You prescribe full antibiotic therapy
- You prescribe antibiotic prophylaxis and, in case of ostectomy/odontotomy , also full antibiotic therapy
